# Supplementary material for: Effective gene expression prediction from sequence by integrating long-range interactions
Source: Nat Methods. 2021 Oct 4;18(10):1196–203. doi: 10.1038/s41592-021-01252-x (PMC8490152; doi:10.1038/s41592-021-01252-x)
Supplement: Supplementary file 1 — Supplementary Methods and Supplementary Figures 1–3 [file 41592_2021_1252_MOESM1_ESM.pdf]

nature portfolio

https://doi.org/10.1038/s41592-021-01252-x

Supplementary information

Effective gene expression prediction from sequence by integrating long-range interactions

In the format provided by the authors and unedited

Supplementary Methods

Experimental-level accuracy

To estimate the experimental-level accuracy of gene expression measurements, we used 204/638 CAGE samples that had multiple replicate experiments (589 experiments in total). For each CAGE sample, we divided the replicate experiments into two groups such that the total number of reads was roughly equal in each group (**Supplementary Table 1**). For each group, we summed the reads for all replicates to generate a pseudo-replicate. Replicate level accuracy was computed by treating data from pseudo-replicate 1 as predictions and data from pseudo-replicate 2 as targets.

Since only 204/638 CAGE samples had multiple replicates, we developed a computational approach to estimate the replicate level accuracy of all 638 CAGE samples. Since many CAGE tracks are highly correlated, a linear combination of other tracks can be used to estimate the replicate experiment of a particular CAGE track. For each track t, we trained a linear model on the same training set as the Enformer model using batch-normalized input values of all other tracks $t' \neq t$ and their log(1+x) transformed version, followed by softplus activation. Performance of this model was evaluated on the same test set as the Enformer model.

Comparison to ExPecto

We obtained the gene expression matrix used by ExPecto from https://github.com/FunctionLab/ExPecto/tree/master/resources. We observed similar test-set median Spearman correlation performance across genes with the pre-trained ExPecto model as reported in the paper (0.812 vs 0.819). We re-trained the Enformer model only on the human genome, holding out chromosome 8, which was used as a test set by ExPecto. We then made predictions with Enformer for all genes, using the sequence centered at the major TSS of the gene as defined by ExPecto. We extracted the relevant CAGE predictions for each gene in two ways: a) by extracting the predictions from the 128 bp bin overlapping the major TSS, b) by averaging the CAGE predictions from all bins overlapping any TSS of the gene as defined by GENCODE v19 annotation. This gave us 638 features for each gene. We quantile normalized these features and trained L2-regularized linear regression on log(1+x) transformed gene expression data from ExPecto training set for all 218 different RNA-seq samples.

TAD boundary attention

We obtained a list of topologically associating domain (TAD) boundaries measured in IMR90 cells following the analysis of Krietenstein et al[1] from https://console.cloud.google.com/storage/browser/basenji_hic/insulation. We randomly selected 1,500 TAD boundaries, extracted the reference genome sequence centered at these positions, made predictions with Enformer, and obtained the transformer attention matrices. We averaged the attention matrices across all sequences, layers, and transformer heads (yielding a

1,536x1,536 matrix). We did the same for 1,500 sequences from our validation set; these sequences do not contain any specific alignment to a particular regulatory element and can hence be considered as ‘background’. We then computed the difference between the two attention matrices (attention_tad_boundary - attention_other) and visualized it as a heatmap. “Query” denotes the position from which we are attending to all other positions in the sequence called “keys”. To compute the statistical significance of the patterns observed in the heatmap, we incrementally partitioned the attention matrix into the following segments:

- Query at insulator: |key| < 6 kb (6kb corresponds to 48 bins of 128 bp)
- Key at insulator: |query| < 6 kb
- Diagonal: |query - key| < 6 kb
- Within TAD: sign(query*key) = 1
- Across TAD: sign(query*key) = -1

Each segment also excludes segments from previous segments thereby making sure that none of the segments overlap (for example “Diagonal” segment will not contain regions overlapping “Key at insulator” and “Query at insulator”). For the attention matrix of each sequence (averaged across layers and heads), we computed the average attention value in the segment of interest.

TAD boundary motif discovery

We computed input*gradient contribution scores for 1,500 sequences centered at TAD boundaries. Gradients were computed with respect to CAGE or DNase targets averaged across all positions and output samples. We ran TF-MoDISco[2] (v0.5.14.0) with default parameters on the obtained CAGE and DNase contribution scores from TAD boundaries. We searched for the closest known motif match in the HOCOMOCO[3] v11 (full) database using Tomtom[4] v4.11.2.

GTEx SLDP detailed description

Signed linkage disequilibrium profile (SLDP) regression is a technique for measuring the statistical concordance between a signed variant annotation v and a genome-wide association study’s marginal correlations $\hat{\alpha}$ between variants and a phenotype[5]. The functional correlation between v and the true variant effects on the phenotype describes how relevant the annotation is for the phenotype’s heritability. Our model produces these signed variant annotations. SLDP estimates this functional correlation using a generalized least-squares regression, accounting for the population linkage disequilibrium structure. It performs a statistical test for significance by randomly flipping the signs of entries in v in large consecutive blocks to obtain a null distribution. We follow previous work in conditioning on background annotations describing minor allele frequency and binary variables for variant overlap with coding sequence (and 500 bp extension), 5′ UTR (and 500 bp extension), 3′ UTR (and 500 bp extension), and introns.

We used GTEx v7a summary statistics for 48 tissues that had been preprocessed for SLDP[6]. In these statistics, each SNP’s effect is summarized over all cis-genes using the following transformation $\hat{\alpha_m} = \frac{1}{\sqrt{|G_m|}} \sum_{k \in G_m} \hat{\alpha}_m^{(k)}$ where G_m is the set of all genes for which a cis-eQTL test was

performed for variant *m* and $\hat{\alpha}_m^{(k)}$ is the marginal correlation of SNP *m* and gene *k* expression[5]. We passed $\hat{\alpha}_m$ to SLDP for analysis of variant predictions.

Dimensionality reduction of variant effect scores

We used principal component analysis (PCA) to reduce 5,313 variant effect features from Enformer to 20 principle components. We used variant effect scores from 1000 Genomes SNPs on chromosome 9 and performed the following steps: (1) subtracted the median and divided by standard deviation estimated from the interquartile range as implemented in RobustScaler in scikit-learn (v0.23.2); (2) reduced the dimensionality to 20 principle components using TruncatedSVD from scikit-learn; and (3) normalized the resulting principal component features using RobustScaler to obtain z-scores.

Supplementary Figures

Supplementary Fig. 1: Attention patterns at the HNRNPA1 locus

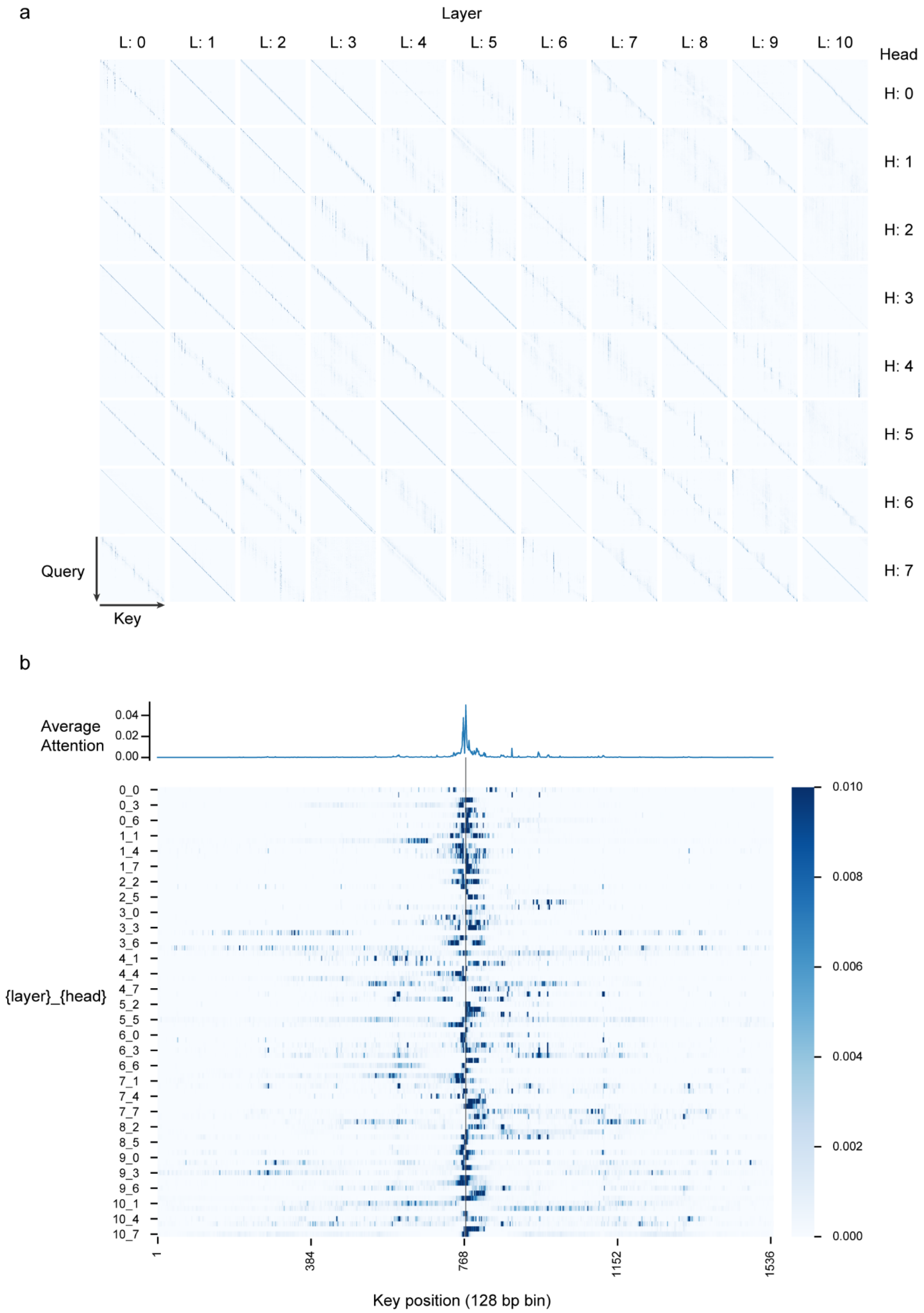

a) Attention matrices for all layers (columns) and heads (rows) for the *HNRNPA1* locus shown in **Fig. 2a**. Most of the attention is placed on the diagonal, meaning that the model is gathering local

information. For some heads, clear vertical lines can be seen (e.g. H: 1, L: 7 or L: 6) meaning that multiple query positions are looking at the same key (such as a gene TSS or enhancer). For some heads, the queries are looking at a specific distance away from the TSS (e.g. H: 6, L: 2). **b)** Attention matrix row from a) where the query position overlaps the TSS of the *HNRNPA1* major TSS stacked for all layers and heads. The average attention across all layers and heads is shown at top and is the same as shown in Fig. 2a and used for enhancer prioritization. Enformer is mostly looking at positions around the TSS in the initial layers (first few rows), with distinct heads looking at positions upstream or downstream of the TSS. In later layers, it starts to look for more distal positions, both using very localized and broad attention.

Supplementary Fig. 2: Example loci from Fulco *et al* 2019

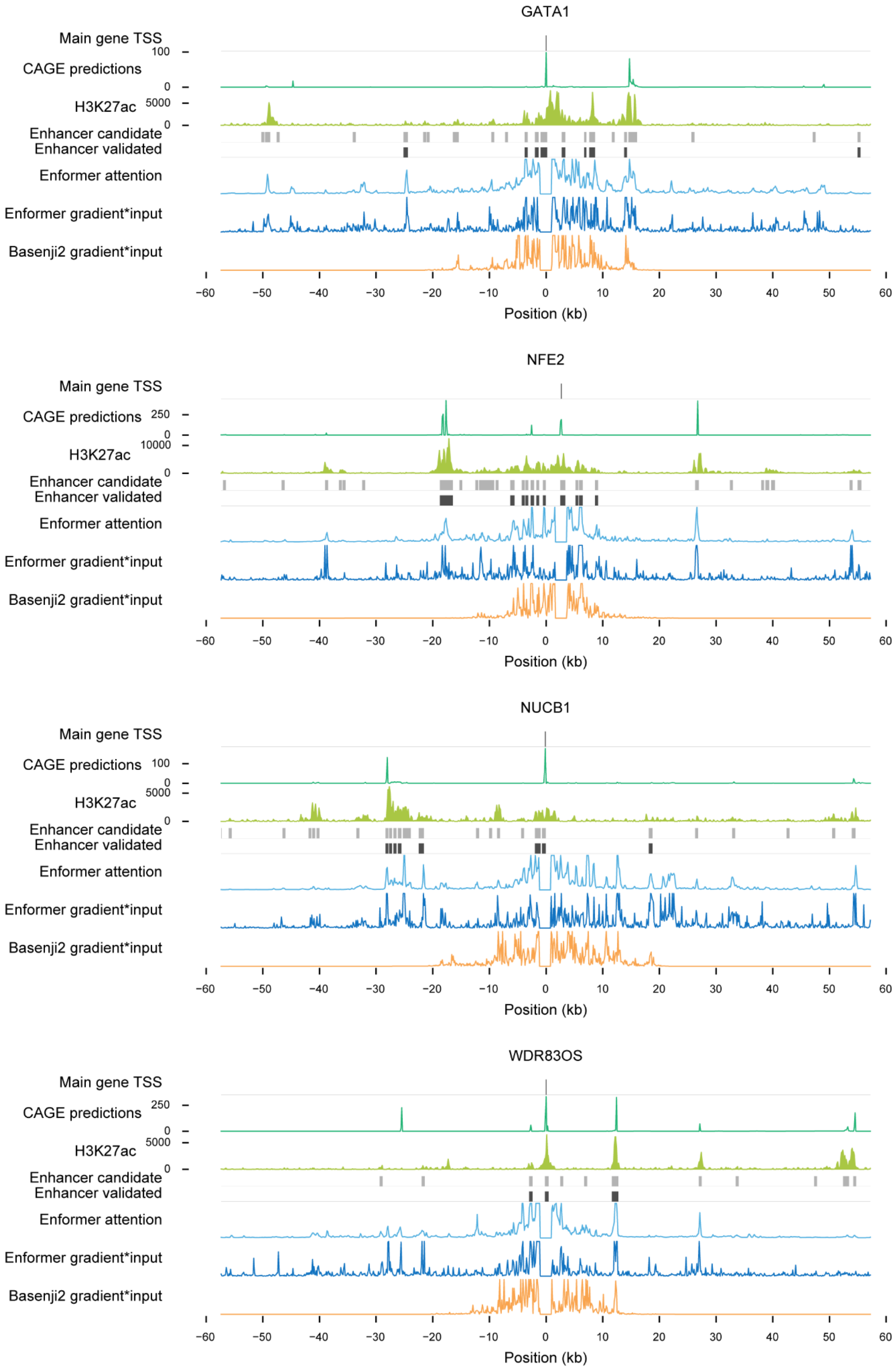

Other example loci from Fulco et al 2019 as shown in Fig. 2.

Supplementary Fig. 3: Example loci from Gasperini *et al* 2019.

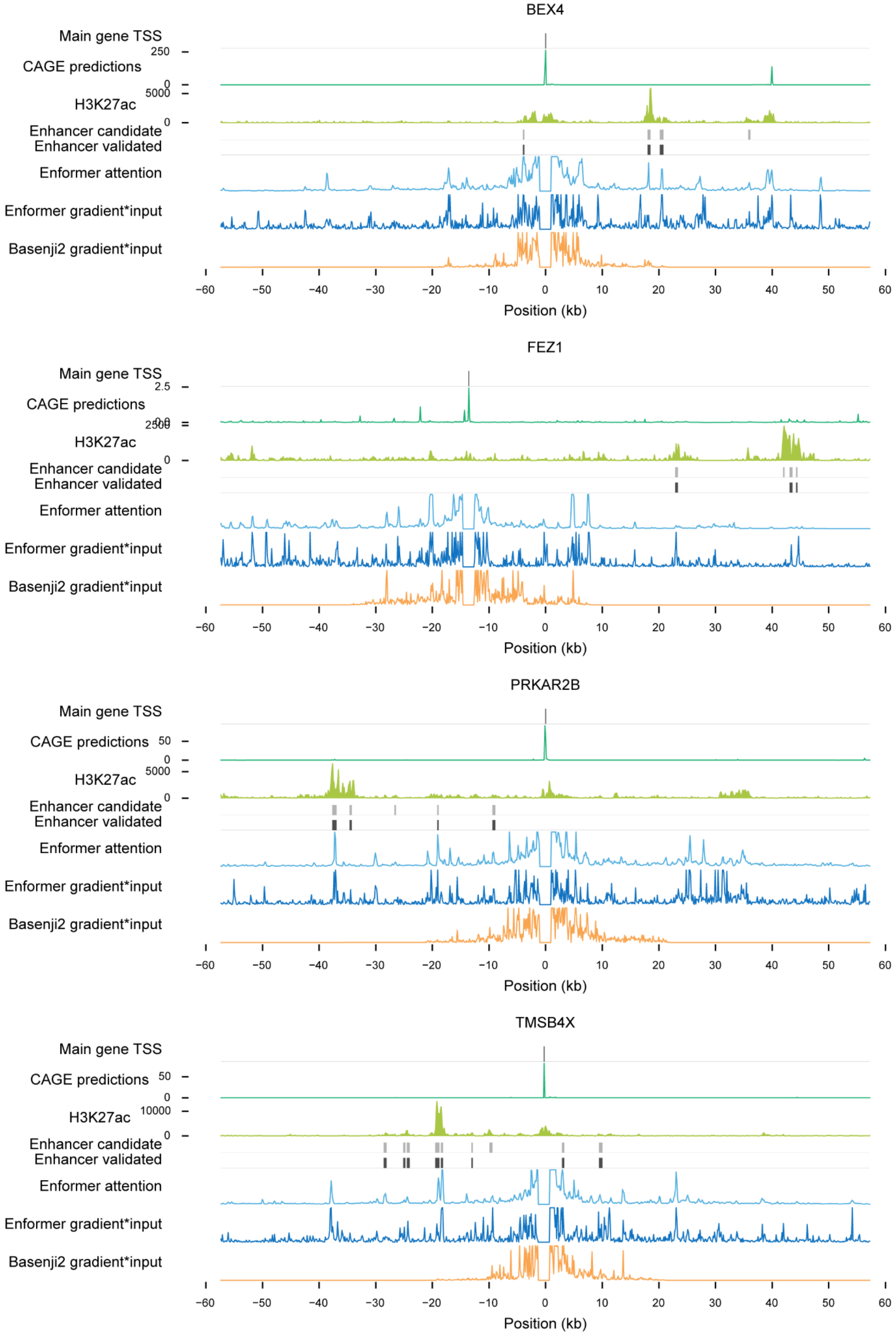

Other example loci from Gasperini et al 2019 as shown in Fig. 2.

References

1. Krietenstein, N. et al. Ultrastructural Details of Mammalian Chromosome Architecture. Mol. Cell 78, (2020).
2. Shrikumar, A. et al. Technical Note on Transcription Factor Motif Discovery from Importance Scores (TF-MoDISco) version 0.5.6.5. (2018).
3. Kulakovskiy, I. V. et al. HOCOMOCO: towards a complete collection of transcription factor binding models for human and mouse via large-scale ChIP-Seq analysis. Nucleic Acids Res. 46, D252–D259 (2018).
4. Gupta, S., Stamatoyannopoulos, J. A., Bailey, T. L. & Noble, W. Quantifying similarity between motifs. Genome Biology vol. 8 R24 (2007).
5. Reshef, Y. A. et al. Detecting genome-wide directional effects of transcription factor binding on polygenic disease risk. Nat. Genet. 50, 1483–1493 (2018).
6. Consortium, T. G. & The GTEx Consortium. The GTEx Consortium atlas of genetic regulatory effects across human tissues. Science vol. 369 1318–1330 (2020).
