# Supplementary material for: Effective gene expression prediction from sequence by integrating long-range interactions
Source: Nat Methods. 2021 Oct 4;18(10):1196–203. doi: 10.1038/s41592-021-01252-x (PMC8490152; doi:10.1038/s41592-021-01252-x)
Supplement: Supplementary file 2 — Reporting Summary [file 41592_2021_1252_MOESM2_ESM.pdf]

nature research

Corresponding author(s): Ziga Avsec, Pushmeet Kohli, David R. Kelley

Last updated by author(s): Jul 13, 2021

Reporting Summary

Nature Research wishes to improve the reproducibility of the work that we publish. This form provides structure for consistency and transparency in reporting. For further information on Nature Research policies, see our Editorial Policies and the Editorial Policy Checklist.

Statistics

For all statistical analyses, confirm that the following items are present in the figure legend, table legend, main text, or Methods section.

n/a	Confirmed	
☐	☒	The exact sample size (*n*) for each experimental group/condition, given as a discrete number and unit of measurement
☒	☐	A statement on whether measurements were taken from distinct samples or whether the same sample was measured repeatedly
☐	☒	The statistical test(s) used AND whether they are one- or two-sided<br>*Only common tests should be described solely by name; describe more complex techniques in the Methods section.*
☐	☒	A description of all covariates tested
☐	☒	A description of any assumptions or corrections, such as tests of normality and adjustment for multiple comparisons
☐	☒	A full description of the statistical parameters including central tendency (e.g. means) or other basic estimates (e.g. regression coefficient) AND variation (e.g. standard deviation) or associated estimates of uncertainty (e.g. confidence intervals)
☐	☒	For null hypothesis testing, the test statistic (e.g. *F*, *t*, *r*) with confidence intervals, effect sizes, degrees of freedom and *P* value noted<br>*Give P values as exact values whenever suitable.*
☒	☐	For Bayesian analysis, information on the choice of priors and Markov chain Monte Carlo settings
☒	☐	For hierarchical and complex designs, identification of the appropriate level for tests and full reporting of outcomes
☐	☒	Estimates of effect sizes (e.g. Cohen's *d*, Pearson's *r*), indicating how they were calculated

Our web collection on statistics for biologists contains articles on many of the points above.

Software and code

Policy information about availability of computer code

Data collection	No software was used for data collection.
Data analysis	All components of our core algorithm, including the full model architecture and example code to train and evaluate the model are available under the open source Apache 2.0 license at the following URL: https://github.com/deepmind/deepmind-research/tree/master/enformer. We used TensorFlow (v2.4.0) and Sonnet (https://github.com/deepmind/sonnet, v2.0.0) to train and evaluate the model. We used the following software in python 3.6 to analyze and visualize data: pandas (v1.1.5), numpy (v1.16.4), scikit-learn (v0.23.2), scipy (v1.2.1), pyBigWig (v0.3.17, https://github.com/deeptools/pyBigWig, git hash: 705b07495ea78740eb7ea94fb0082e23661d9929), pyranges (https://github.com/biocore-ntnu/pyranges, v0.0.76), nucleus (https://github.com/google/nucleus, v0.5.8), TF-MoDISco (v0.5.6.5), seaborn (v0.11.1), matplotlib (v3.0.3), and plotnine (v0.7.1). We also used the UCSC liftOver tool (Jan 2020 release).

For manuscripts utilizing custom algorithms or software that are central to the research but not yet described in published literature, software must be made available to editors and reviewers. We strongly encourage code deposition in a community repository (e.g. GitHub). See the Nature Research guidelines for submitting code & software for further information.

Data

Policy information about availability of data

All manuscripts must include a data availability statement. This statement should provide the following information, where applicable:

- Accession codes, unique identifiers, or web links for publicly available datasets
- A list of figures that have associated raw data
- A description of any restrictions on data availability

Gene annotation was obtained from https://www.gencodegenes.org/ (v32). Basenji2 training, validation, and test data was obtained from https://console.cloud.google.com/storage/browser/basenji_barnyard/data. Processed CRISPRi data for Fulco et al 2019 was obtained from supplementary material and for

Gasperini et al 2019 from GEO accession GSE120861. H3K27ac ChIP-seq data in K562 used for analysis in Fig. 2 was obtained from https://www.encodeproject.org/ with file accession ENCFF779QTH and DNase with file accessions ENCFF413AHU and ENCFF936BDN. TAD boundaries processed by Fudenberg et al 2020 were obtained from https://console.cloud.google.com/storage/browser/basenji_hic/insulation. Fine-mapped eQTLs are available from the supplementary material of Wang et al 2021. We acquired training and test sets as well as the predictive accuracies of individual competition participants from the CAGI5 competition (M. Kircher, personal communication, https://genomeinterpretation.org/content/expression-variants). For comparison to ExPecto, we used the provided data from https://github.com/FunctionLab/ExPecto/tree/master/resources.

Field-specific reporting

Please select the one below that is the best fit for your research. If you are not sure, read the appropriate sections before making your selection.

☒ Life sciences ☐ Behavioural & social sciences ☐ Ecological, evolutionary & environmental sciences

For a reference copy of the document with all sections, see nature.com/documents/nr-reporting-summary-flat.pdf

Life sciences study design

All studies must disclose on these points even when the disclosure is negative.

Sample size	We used 5,313 human datasets and 1,643 mouse datasets to train and evaluate the Enformer model (Supplementary Table 2,3). This dataset (and therefore the number of samples) was compiled and already used by the previous best model (Basenji2).
Data exclusions	No data were excluded from analyses.
Replication	No experimental findings were disclosed, hence no replication was performed.
Randomization	Randomization was not relevant for this study as it involved a reanalysis of published datasets.
Blinding	Not applicable. No new data were collected.

Reporting for specific materials, systems and methods

We require information from authors about some types of materials, experimental systems and methods used in many studies. Here, indicate whether each material, system or method listed is relevant to your study. If you are not sure if a list item applies to your research, read the appropriate section before selecting a response.

Materials & experimental systems

n/a	Involved in the study	
☒	☐	Antibodies
☒	☐	Eukaryotic cell lines
☒	☐	Palaeontology and archaeology
☒	☐	Animals and other organisms
☒	☐	Human research participants
☒	☐	Clinical data
☒	☐	Dual use research of concern

Methods

n/a	Involved in the study	
☒	☐	ChIP-seq
☒	☐	Flow cytometry
☒	☐	MRI-based neuroimaging
